# Relationship between *Feline calicivirus* Load, Oral Lesions, and Outcome in Feline Chronic Gingivostomatitis (Caudal Stomatitis): Retrospective Study in 104 Cats

**DOI:** 10.3389/fvets.2017.00209

**Published:** 2017-12-05

**Authors:** Isabelle Druet, Philippe Hennet

**Affiliations:** ^1^ADVETIA Veterinary Specialty Center, Paris, France

**Keywords:** stomatitis, gingivostomatitis, caudal stomatitis, calicivirus, viral load, dental extractions, dentistry, cats

## Abstract

**Objective:**

This study was performed to assess the relationship between oral *Feline calicivirus* (FCV) load and severity of lesions at the time of presentation of cats suffering from feline chronic gingivostomatitis (FCGS) (part 1) and treatment outcome after dental extractions (part 2). We hypothesized that a higher FCV viral load would be positively correlated with the severity of lesions at presentation and negatively correlated with treatment outcome. In addition, the effect of dental extractions on outcome and the influence of preoperative severity of lesions on the outcome were investigated.

**Materials and methods:**

Cats with FCGS were included in the study if they had been diagnosed with caudal stomatitis, had been tested positive for FCV using a real-time reverse transcriptase-PCR test on oropharyngeal swab, and had dental extractions performed within the authors’ department. General practitioners provided all previous medical treatments. Cats with recheck examinations were included in part 2 of the study. Multivariate statistical analysis was performed to assess the relationship between the different parameters.

**Results:**

One hundred four cats met the requirements for part 1 and 56 cats for part 2 of the study. Data collected from patients’ record included patient history, viral testing results, extent and severity of oral lesions, extent of teeth extraction. Signalment, history, preoperative treatment, and severity of caudal and alveolar stomatitis score were not associated with FCV load (*P* > 0.05). Presence of lingual ulcers was significantly correlated with FCV load (*P* = 0.0325). Clinical cure (32.1%) or very significant improvement (19.6%) was achieved in 51.8% of cats within 38 days. Concomitantly, 60.7% of the owners considered their cat cured (41.1%) or significantly improved (19.6%). Extent of teeth extraction was not found to influence the clinical outcome (*P* > 0.05).

**Conclusion:**

The results of this study did not support the hypothesis that FCV oral load is correlated with the severity of oral lesions or with the outcome following dental extractions. In addition, the severity of caudal inflammation was not correlated with healing time or achievement of cure.

## Introduction

Feline chronic gingivostomatitis (FCGS) is a painful and debilitating feline oral condition characterized by chronic severe bilateral inflammation of the gingiva, alveolar, labio-buccal mucosa, and caudal oral mucosa (1–4). Ulcerative or ulceroproliferative lesions are often observed. Ulceration of the tongue and palate may also be present. In addition, FCGS has been shown to be associated with more widely distributed and severe periodontitis and with a higher prevalence of external inflammatory root resorption and retained roots than other oral diseases (2). The presence of caudal stomatitis distinguishes FCGS from other feline oral conditions (1, 2, 5). Cats affected by FCGS are often presented with dysorexia/anorexia, oral pain, weight loss, ptyalism, halitosis, and lack of grooming (3, 6–8).

Histological findings include a lymphoplasmacytic infiltration of the mucosa and the submucosa (9–12). The pattern and distribution of oral lesions is typical and a presumed diagnosis can be reached based solely on the clinical findings (4, 13). The condition results from an inappropriate response of the host’s immune system to chronic oral antigenic stimulation of various origins and is considered multifactorial (1, 4). The inflammatory response, showing an increased T-lymphocyte count compared with B-lymphocyte numbers, suggests that this condition may, in fact, be associated with viral infections (9).

Different infectious and non-infectious causes have been suspected: bacteria (*Pasteurella multocida, Bartonella* sp.), viruses (feline immunodeficiency virus (FIV), feline leukemia virus (FeLV), feline herpesvirus (FHV-1), and *Feline calicivirus* (FCV), dental diseases, and allergic reactions) (7, 14–17). FCV has been identified as a causative agent of upper respiratory tract infection and ulcerative lesions in the oral cavity. However, it may also be detected in cats without clinical signs (15, 18–20). Though no direct causal relationship between FCV and FCGS has been established, various experiments support its involvement (15, 17, 19). Acute caudal stomatitis has been experimentally induced in germ-free cats with inoculation of FCV strains sampled from the oropharynx of cats suffering from chronic stomatitis (21). The seropositive prevalence of FCV has been reported to be higher in FCGS, with almost 100% of positive cats, than in the general population (7, 14, 17, 19, 22).

Medical treatment alone is unrewarding and has been shown to only provide temporary improvement (3, 4, 23). Extraction of all teeth or premolar and molar teeth is the currently accepted standard of care, with similar results between full-mouth and premolar–molar extractions (3–5, 8). Substantial improvement or complete remission has been reported in 67–80% of FCGS cats (3, 5, 8). Nevertheless, 69% of cats showing improvement still required extended medical treatment (3). Cats showing little or no improvement (refractory cases) also required continuous medical treatments. Recent studies have shown that oromucosal administration of interferon omega (1), oral administration of cyclosporine (24), and IV injection of stem cells (11, 25) may provide some improvement or even a cure for refractory cats.

Progress in molecular biology has led to the development of quantitative polymerase chain reaction techniques (real-time PCR), which enable evaluation of the viral load in the oral cavity. Decrease of FCV load has been shown to be significantly correlated with clinical improvement and oromucosal ulcer scores in a prospective study of FCV-positive cats suffering from feline upper respiratory tract disease (FURTD) and treated by local interferon omega administration (26). Furthermore, a decrease of FCV load was reported in a cat suffering from FCGS and shedding FCV that was successfully treated by dental extraction followed by interferon omega therapy (27).

The aim of this study was to investigate the relationship between FCV load and severity of FCGS before (study part 1) and after dental extractions (study part 2). We hypothesized that cats with higher FCV load would present with more severe lesions.

In addition, the outcome following dental extractions and the influence of preoperative severity of lesions on the outcome were studied.

## Materials and Methods

### Case Selection

Medical records of cats examined in the Dental and Oromaxillofacial Department of Advetia Veterinary Referral Center, Paris, France, between January 2011 and February 2016 were searched for the followings keywords: stomatitis, calicivirus. The following inclusion criteria were required: presence of FCGS with caudal stomatitis (inflammation of the caudal oral mucosa lateral to the palatoglossal folds), FCV positivity on real-time reverse transcriptase-PCR (RT-PCR) test and no extraction performed before appointment.

All cats had had oropharyngeal swabs collected under general anesthesia using a cytobrush (GIMA brush, Gessate, Italy). Each sample was preserved in a sterile glass serum tube (BD Vacutainer, Plymouth, UK) and transported to the Analysis Department of Scanelis Laboratory, France to detect FCV RNA by real-time RT-PCR. A real-time RT-PCR designed in the conserved 5° region of the viral genome was used to detect FCV RNA as described previously (28). Detection level was 200 copies per sample. Based on the laboratory recommendations, a cutoff titer of 10e4 was used to distinguish between low and high FCV load.

Comprehensive oral and dental examination with full-mouth intraoral radiographs was performed before surgical treatment. Full mouth extraction (FME), subtotal mouth extraction (SME), which includes a least all premolar and molar teeth but not all teeth, or partial mouth extraction (PME), which includes extraction of a selective number of teeth, were performed depending on individual criteria. Extraction was performed for teeth associated with ulcerative gingivitis as well as alveolar and/or buccal stomatitis, teeth presenting with dental resorption, periodontitis, or pulpal pathology. Professional scaling and polishing were performed for the remaining teeth. Preoperative and postoperative analgesics methadone (Comfortan, Dechra Veterinary Products SAS, Montigny le Bretonneux, France) or buprenorphine (Bupaq, Virbac, Carros, France) ± meloxicam (Metacam; Boehringer Ingelheim, Rheims, France) and 2 weeks of postoperative antibiotic therapy (ABT) (clindamycin: Antirobe; Zoetis, Malakoff, France) were administered. Cats presenting with concomitant diseases (e.g., oral tumor, organic, or metabolic diseases) or those with incomplete records were excluded from the study.

### Medical Record Review

Signalment (breed, gender, neuter status, age, and weight), medical history (viral status for FeLV, FIV, and FVH-1, history of FURTD, duration of symptoms of stomatitis, and previous medical management), and clinical findings (distribution and severity of oral lesions, presence of lingual ulcerations, size of mandibular lymph node at palpation) were recorded. Oral inflammatory lesions (caudal stomatitis and alveolar/buccal stomatitis) were graded by the same operator (PH) according to a modification of a previously described scoring system (Table 1) (1). A global caudal stomatitis intensity score (GCSIS) was calculated using the formula: (caudal intensity score × surface area score)/100.

**Table 1:** Scoring systems used for evaluation of oral lesions.

*Caudal stomatitis intensity score and alveolar stomatitis intensity score (ASIS)* 0: Absence of lesion 1: Slight inflammation. No ulceration. No proliferation. No spontaneous bleeding. No bleeding induced by gentle pressure 2: Mild inflammation. No ulceration. No or slight proliferation. No spontaneous bleeding. No bleeding induced by gentle pressure 3: Moderate inflammation. Possible ulcerative or ulceroproliferative lesion. No spontaneous bleeding but bleeding induced by gentle pressure on the lesions 4: Severe inflammation. Possible ulcerative or ulceroproliferative lesion. Spontaneous bleeding
*Surface area score for caudal stomatitis* Inflammatory lesions are scored on both sides as: 0 for absence of lesion 25 for ≤25% of the total surface area 50 for 25–50% of the total surface area 75 for 50–75% of the total surface area 100 for ≥75% of the total surface area

Follow-up visits were divided into eight time periods: 0–25, 26–50, 51–75, 76–100, 101–125, 126–150, 151–250, and over 250 days. When the cat was cured (no sign of pain and no oral lesion), follow-up visits were discontinued.

At each follow-up visit, the following criteria were recorded: scoring of oral inflammation, lymph node enlargement (normal = score 0; mild enlargement = score 1; marked enlargement = score 2), final outcome (cured = score 1; not cured = score 0), and owner’s satisfaction (score 0: no improvement, score 1: little improvement, score 2: significant improvement and score 3: cure).

### Statistical Analysis

Both descriptive and analytical statistical analyses were performed with a commercial software (Statgraphics Centurion version XVI.II, DYNACENTRIX, Neuilly sur Seine, France). The statistical influence of categorical factors FCV load on the severity of FCGS lesions was analyzed. The relationship between those factors and oral lesions or lymph node enlargement was assessed using a simple linear regression, respectively. The statistical influence of those factors on lingual ulceration was evaluated using a logistic regression. The evolution of clinical cure rates over time depending on several risk factors [FCV load, GCSIS, and alveolar stomatitis intensity score (ASIS)] at initial assessment was analyzed by means of Kaplan–Meier method and Cox model. A threshold value of α = 0.05 was used to define significance.

## Results

Initial review of cases identified 147 cats suffering from FCGS and tested for FCV by RT-PCR. 104 cats met the inclusion criteria for part 1 of the study (relationship between FCV load and severity of oral lesions before treatment). Among this population, 56 cats had at least one post-extraction follow-up visit performed in our department and were included in part 2 of the study (relationship between FCV load and outcome). Cats which had follow-up visits performed at the referring veterinary practice were excluded.

Patient’s features and viral status are summarized in Table 2. Clinical findings, lingual ulcerations, lymph node enlargement, lesional scores, and treatments before initial evaluation are compiled in Table 3. Duration of clinical signs was documented for 86 (81%) cats and the median time was 12 months (range 0.5, 60 months) (mean 14.6 ± 12.8 months). Cats presented a FCV load ranging from 200 to 1.2 × 10e6 copies per sample with 45/104 (43.3%) cats showing a load inferior to 10e4 copy by sample and 59/104 (56.7%) a load superior to 10e4 copy by sample.

**Table 2:** Characteristics and viral status of *Feline calicivirus*-positive cats.

	Part 1 (*n* = 104)	Part 2 (*n* = 56)
**Breed [number (%) of cats]**		
Domestic shorthair cat	85 (81.6)	46 (82.1)
Maine coon	6 (5.7)	3 (5.4)
Norwegian	4 (3.8)	3 (5.4)
Oriental	3 (2.9)	2 (3.6)
Chartreux	2 (2.8)	1 (1.8)
Birman	1 (0.9)	1 (1.8)
Siamese	1 (0.9)	–
Turkish Angora Cat	1 (0.9)	–
British Shorthair	1 (0.9)	–
**Gender [number (%) of cats]**		
Sexually intact female	–	–
Spayed female	40 (38.8)	21 (37.5)
Sexually intact male	4 (3.8)	4 (7.1)
Castrated male	60 (57.6)	31 (55.4)
**Age (in years)**		
Mean age ± SD	7.26 ± 4.0	7.47 ± 4.3
**Viral status [number (%) of cats]**		
Tested for feline immunodeficiency virus (FIV)	89/104 (85.4)	49/56 (87.5)
FIV positive	12/89 (13.5)	6/49 (12.2)
Feline leukemia virus positive	0/89 (0)	0/49 (0)
Tested for FHV	80/104 (76.8)	43/56 (76.8)
FHV positive	14/80 (17.5)	7/43 (16.3)

*Part 1: population at the first assessment, part 2: sub-population with follow-ups*.

**Table 3:** Medical management before the first assessment and clinical findings at the first assessment (D0).

	No. (%) of cats
**Medical management before first appointment**	
SAIDs	19/93 (20.4)
NSAIDs	9/93 (9.7)
Antibiotic therapy (ABT)	5/93 (5.4)
SAIDs + ABT	25/93 (28.9)
NSAIDs + ABT	12/93 (12.9)
SAIDs + NSAIDs	4/93 (4.3)
SAIDs + NSAIDs + ABT	4/93 (4.3)
No treatment	15/93 (16.1)
History of feline upper respiratory tract disease	35/68 (51.7)
**Clinical finding**	
Mandibular lymph node palpation	93/104 (89.4)
Normal	1/93 (1.1)
Mild enlargement	61/93 (65.6)
Marked enlargement	32/93 (34.4)
Lingual ulcer	15/51 (29.4)
**Lesional score**	
GCSI	
0 to <1	9/104 (8.6)
1 to <2	29/104 (27.9)
2 to <3	27/104 (26.0)
3 to <4	33/104 (31.7)
4	6/104 (5.8)
ASIS	
0/4	3/95 (3.1)
1/4	4/95 (4.2)
2/4	18/95 (18.9)
3/4	59/95 (62.1)
4/4	11/95 (11.5)

Presence of lingual ulcers was significantly correlated with FCV load (*P* = 0.0325). Cats with a high FCV load had more risk to present a lingual ulcer. Caudal stomatitis score and alveolar stomatitis score were not associated with FCV load (*P* = 0.5150 and *P* = 0.1719, respectively). Severity of preoperative oral lesions was not correlated to signalment, history, or preoperative medical treatment (*P* > 0.05).

**Figure 1:**
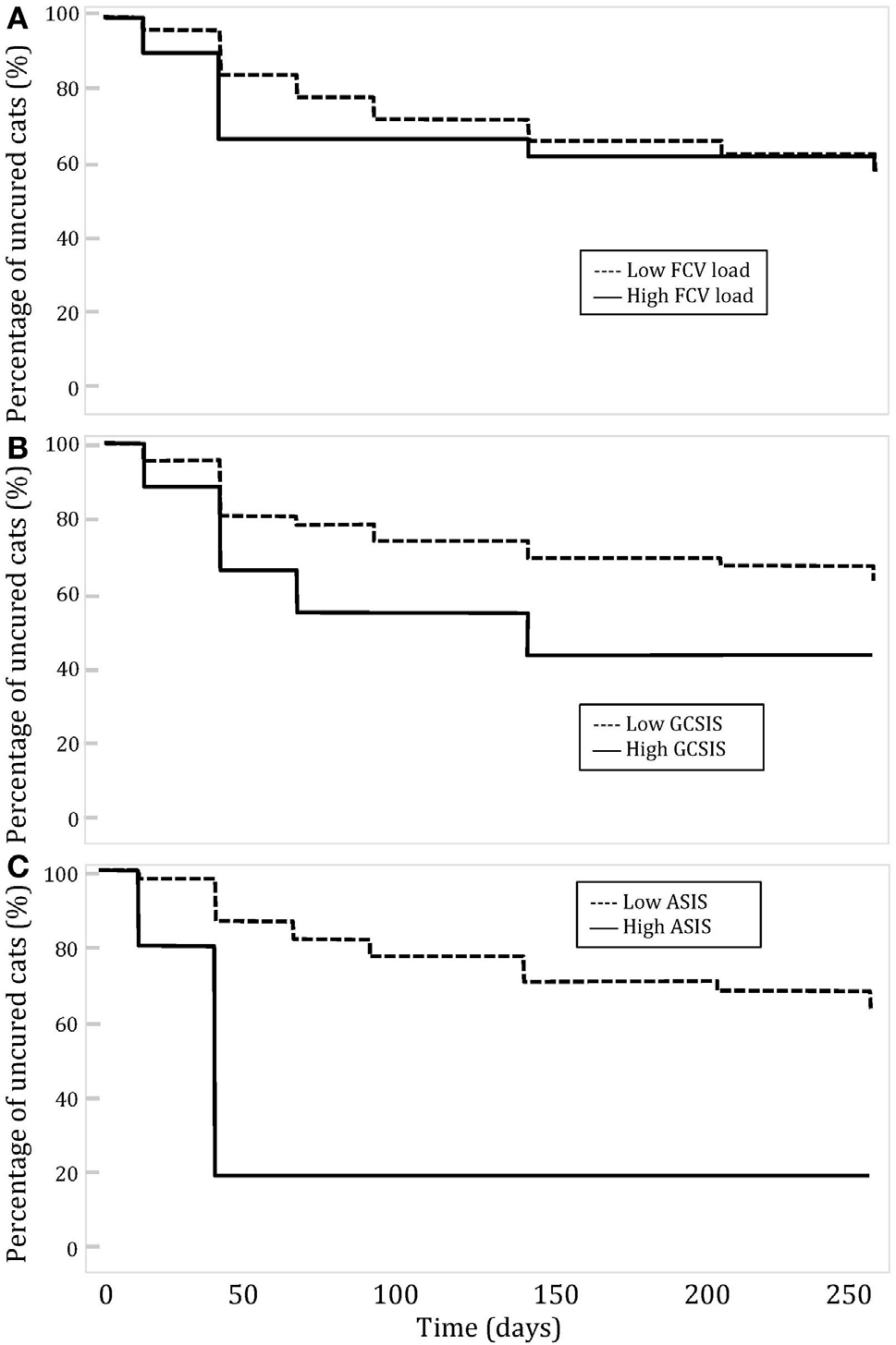
Evolution of clinical cure over time in cats with feline chronic gingivostomatitis depending on **(A)** *Feline calicivirus* (FCV) load; **(B)** global caudal stomatitis intensity score (GCSIS) at the first assessment; and **(C)** aveolar stomatitis intensity score (ASIS) at the first assessment.

Fifty-six cats (53.8%) cats had at least one follow-up visit performed in our service. Epidemiological characteristics and signalment of this subpopulation were similar to the initial studied population (Table 2). The relationship between clinical improvement, preoperative FCV load, and preoperative clinical lesions: GCSIS and ASIS was studied. Major clinical improvement was considered when postoperative scores decreased by 50% or more and slight improvement when the scores decreased by less than 50%. A high FCV load was assigned to cats with a titer superior to 10e4 copies per sample and a low FCV load for cats with a titer inferior to 10e4 copies per sample. Kaplan–Meier curves showing the evolution of clinical cure depending on FCV load, GCSIS, and ASIS at first assessment are shown on Figure 1.

No significant relation was observed between clinical improvement and low or high FCV load or severity of caudal lesion (GCSIS). Cats with a low FCV load or a low severity of oral lesions did not improve faster than cats with a high load or more severe lesions. Cats with low ASIS improved significantly more rapidly than cats with high ASIS (*P* = 0.0294). Clinical lesion scores at each follow-up period were compared to preoperative scores (Table 4). The mean value of GCSIS during the first 100 days post-surgery was significantly lower than preoperative scores. The mean value of ASIS was significantly different in groups T2, T4, and T6. The mean value of lymph node involvement was significantly different for the group T2, T4, T7, and T8.

**Table 4:** Evolution of scores after surgical treatment compared to the first assessment (D0).

	Global caudal stomatitis intensity score	ASIS	Lymph node involvement
T1 < 25 days	***P* = 0.0159**	*P* = 0.0736	*P* = 0.6171
T2 = 25–50 days	***P* = 0.00001**	***P* = 0.0015**	***P* = 0.0026**
T3 = 51–75 days	***P* = 0.0455**	*P* = 0.07364	*P* = 0.0736
T4 = 76–100 days	***P* = 0.0033**	***P* = 0.0133**	***P* = 0.0455**
T5 = 101–125 days	*P* = 0.7237	*P* = 0.6171	*P* = 1.0000
T6 = 126–150 days	*P* = 0.2207	***P* = 0.0233**	*P* = 0.1336
T7 = 151–250 days	*P* = 0.0961	*P* = 0.3711	***P* = 0.0233**
T8 > 251 days	*P* = 0.1138	*P* = 0.2207	***P* = 0.0412**

*In bold: significant results (P < 0.05)*.

Surgical treatment consisted of surgical dental extraction according to the severity of inflammation and dental infections: 3/56 cats underwent FME, 47/56 SME, and 6/56 cats underwent PME. The type of dental extraction was not found to influence the clinical outcome. Clinical cure was defined as absence of pain and completely healed oral lesions. It was observed in 18/56 (32.1%) of cats within a median of time of 33.5 days (range 14, 189 days) (mean 63.3 ± 53.8 days). Nine of these cats (50%) did not receive any medication beyond the first 2 weeks of the postoperative period and were clinically cured within 28 ± 5.8 days (median 27.7; range 22, 38 days). Persistence of oral lesions and some degree of pain were observed in 38/56 (67.9%) cats among which 11 cats (28.9%) did not require further medical treatments beside oral antiseptics in a few cases (Table 5). Median time to achieve that status in these 11 cats was 49 days (range 21, 228 days) (mean 73.6 ± 71.6 days).

**Table 5:** Owner and clinical outcome.

Owner outcome		Clinical assessment	
	No (%) of cats		No (%) of cats
No improvement	4 (7.1)	No cure	38 (67.9)
Little improvement	18 (32.1)	– *medical treatment*	*27 (71.1)*
Significant improvement	11 (19.6)	– *no medical treatment*	*11 (28.9)*
Cure	23 (41.1)	Cure	18 (32.1)

Owner’s satisfaction was subjectively and globally based on cat’s wellness, ability to eat wet or dry food, signs of pain, and grooming activity. Four cats out of 56 (7.1%) were considered not improved, 18/56 (32.1%) slightly improved, 11/56 (19.6%) significantly improved, and 23/56 (41.1%) were considered cured. Three cats (5.4%) were euthanized due to severe oral pain 333 ± 76 days after dental surgery. A significant correlation was observed between owner’s satisfaction and clinical cure (*P* < 0.0001).

At the last follow-up, 19/56 (33.9%) cats did not receive any treatment, and 9/56 (16.6%) cats only received regular local treatment: chlorhexidine gel (Elugel; Pierre Fabre Médicament, Boulogne, France). Various medical treatments, in combination or alone, were administrated in 28/56 (50.0%) cats: oromucosal interferon omega (Virbagen Omega; Virbac, Carros, France) was administrated in 12/56 (21.4%) cats; non-steroidal-anti-inflammatory drugs; meloxicam: (Metacam; Boehringer Ingelheim, Rheims, France) in 8/56 (14.8%) cats, antibiotic treatment; clindamycin (Antirobe; Zoetis, Malakoff, France) or amoxicillin–clavulanic acid (Kesium; CEVA Santé animale, Libourne, France) in 6/56 (10.7%) cats, steroidal-anti-inflammatory drug; prednisolone (Dermipred; CEVA Santé animale, Libourne, France) in 5/56 (8.9%) cats and oral cyclosporine (Atopica; Elanco Europe, Basingstoke, UK) in 5/56 (8.9%).

## Discussion

This retrospective study of cats suffering from FCGS investigated the relation between FCV load, oral lesions, and outcome. The cats’ characteristics in our population were similar to those of previous studies (2, 3, 29). The median age of the population was 7.3 years (range 1, 19 years) (mean 7.5 ± 4.0 years), and this distribution was similar to previous reports (1–3, 5, 6, 19). Cats included in this study had been long suffering from stomatitis with a median time of 12 months (range 0.5–60 months) (mean 14.6 ± 12.7 months), which is slightly longer than reported in a previous retrospective study (3). This long delay is indicative of how frustrating and difficult the management of this condition by general practitioners can be and of the late decision to consider dental extractions.

No cat was detected positive for FeLV whereas 13.5% were FIV positive. A large scale epidemiological study in North-America compared seropositivity of cats with oral diseases with that of general population; a seropositivity of 3.1% for FeLV and 3.6% for FIV was found in general population whereas a higher prevalence was found in the population of cats presenting with oral diseases: 4.7% were positive for FeLV and 9.7% for FIV (30). Studies in New Zealand and Australia have also shown a lower prevalence of FeLV compared to FIV (31, 32). In a retrospective study on feline caudal stomatitis, no cat was seropositive for FeLV and 4.1% of the cats were positive for FIV (3). In the UK, a study has shown similar levels of seropositivity in cats housed in different shelters but that seroprevalence may vary with populations (33). Our results are in accordance with these reports.

Feline herpesvirus is commonly associated with FURTD (34). Viral shedding is intermittent and the virus may stay latent in trigeminal ganglia making detection by RT-PCR on oropharyngeal swab uncertain (35). A Spanish study in a population of 358 cats has shown FHV-1 carriage in 28.3% of cats with FURTD, 15.3% of cats with oral lesions, and 6% of healthy cats (36). In a study in cats affected by FCGS, presence of FHV-1 was not significantly different in diseased cats (13.5%) and in controls (6.0%) (19). We tested 17.5% (14/80) cats positive for FHV-1, which is in accordance with the previous studies.

Cats included in this study were referred for the treatment of FCGS and showed severe oral inflammatory lesions; 63.5% of them had a GCSIS greater than 2 out of 4 and 73.6% of them had an ASIS greater than 3 out of 4. Mild mandibular lymph node enlargement was noticed in 65.6% of the cats and severe enlargement in 34.4% of them. Direct comparison with other studies is not possible as there is no standard scoring system for this condition. A low FCV load (<10e4 copies per sample) was found in 43.3% of the cats and a high FCV load was found in 56.7% of the cats. Nevertheless, no significant relation was found between FCV load and severity of oral lesions (assessed through GCSIS or ASIS). A significant correlation (*P* = 0.0325) was found between FCV load and presence of lingual ulcers. In a previous report, oral ulcerations including lingual ulcers were significantly associated with FCV-positive cats (20). However, in that same study though FCV load was higher in diseased cats than in healthy cats, statistical significance was not reached (20). Based on the results of this study, it cannot be speculated that cats with a higher FCV load present with more severe oral ulcerative lesions or that cats with severe oral lesions have a higher viral load. No other signalment or history parameter was correlated with severity of oral lesions. Interestingly, duration of clinical signs was not correlated to clinical scores. Because of the case selection and referral practice nature, median duration of clinical signs at time of referral was 12 months (range 0.5, 60 months) (mean 14.6 ± 12.7 months); lesions may have been at that time already so severe that differences were not significant anymore. At time of referral, cats were receiving various treatments including SAID (56%), antibiotics (49.5%), and NSAID (31%). The type of preoperative treatment was not found to significantly influence the severity of oral lesions. This is in agreement with previous reports, which reported that no medical treatment is found superior to another (37, 38).

Because of the referral nature of our practice, postoperative examination was performed at the general practitioner’s practice for some cats and only 56 cats could be included in the second part of this study. SME was performed in 84%, PME in 11%, and FME in 5% of the cats. No statistical difference was found between the three techniques but the low numbers in the two latter categories may have resulted in a low statistical power. Nevertheless, a recent retrospective study did not show any differences in outcome between SME and FME (3). Most clinical scores significantly improved within the first 100 days confirming the positive effect of dental extractions in cats suffering from FCGS (3, 5, 8). After that period, differences were scarce and might be explained by the fact that cats needing recheck examination beyond 100 days post-operatively were mostly refractory cases showing little or no clinical improvement.

Full mouth or SME is considered to be the current standard of care for FCGS with approximately 70–80% of cats showing substantial improvement or complete remission (3–5, 8). In our cohort, 18/56 (32.1%) cats were clinically cured after extractions (no persistent clinical signs or lesions) and 11/56 (19.6%) did not require any medical treatment beyond chlorhexidine topical treatment, though they still showed some level of inflammation. Overall, 29/56 (51.8%) showed significant improvement or cure within a median time of 38 days (range 14, 228 days) (mean 67.3 ± 60.0 days). Accordingly, the owners reported a high satisfaction rate: 34/56 (60.7%) cats were considered significantly improved or cured. This owner’s satisfaction rate was significantly correlated with clinical improvement and cure (*P* < 0.0001). These results confirm those of previously published studies.

We were not able to confirm the hypothesis that FCV load might have an influence on the post-extraction outcome. No significant relationship was observed between clinical improvement and low or high FCV load or severity of caudal lesion (GCSIS). Cats with a low FCV load or with low-grade caudal oral lesions did not improve faster than cats with a high load or more severe lesions. However, cats with low ASIS improved significantly more rapidly (*P* = 0.0294) than cats with high ASIS. Though it may seem logical that cats presenting with slight alveolar/buccal inflammation have a better outcome than cats with severe lesions, it is difficult to explain the discrepancy between caudal stomatitis and alveolar/buccal stomatitis in this regard. It may be that the presence of caudal stomatitis is more important than its intensity and/or that alveolar/buccal stomatitis is a more significant factor than caudal stomatitis. However, this does not reflect our personal experience. Sample size may not have been large enough to demonstrate a difference for caudal stomatitis, though a difference was seen for alveolar/buccal stomatitis.

This study had some limitations, the most important one being its retrospective nature. Furthermore, the referral nature of the practice has resulted in some loss in follow-ups and impossibility to reevaluate lesions. Even though the outcome of lost cases was reported by the owner or the general practitioner, these cases were not reported in this study.

## Conclusion

This study showed that severity of lesions in cats with FCGS and outcome after dental extractions are not related to FCV load. In addition, FCV load and severity of lesions were not correlated with the time necessary to achieve improvement. Nevertheless, cats presenting with less severe alveolar/buccal stomatitis improved significantly more rapidly. The beneficial effect of dental extractions was confirmed. Clinical cure (32.1%) or very significant improvement (19.6%) was achieved in 51.8% of cats within 38 days. Concomitantly, 60.7% of the owners considered their cat as cured (41.1%) or significantly improved (19.6%).

## Author Contributions

All authors listed have made a substantial, direct, and intellectual contribution to the work and approved it for publication.

## Conflict of Interest Statement

PH and ID are employed at ADVETIA Veterinary Specialty Center. The reviewer PP and handling editor declared their shared affiliation.
